# Primary tracheal synovial sarcoma: a rare clinical entity with diagnostic challenges

**DOI:** 10.1186/s43046-019-0014-z

**Published:** 2020-01-10

**Authors:** Navin Kumar, Seema Kaushal, Kanaklata Kanaklata, Manoj Gowda, Sunil Kumar

**Affiliations:** 1 Department of Surgical Oncology, Institute Rotary Cancer Hospital, All India Institute of Medical Sciences (AIIMS), F 23, Ansari Nagar (West), New Delhi, 110029 India; 2 Department of Pathology, All India Institute of Medical Sciences (AIIMS), New Delhi, 110029 India; 3 Department of Nuclear Medicine, All India Institute of Medical Sciences (AIIMS), New Delhi, 110029 India

**Keywords:** Tracheal tumors, Synovial sarcoma, Tracheal resection, Tracheoplasty

## Abstract

**Background:**

The incidence of primary tracheal tumors is very low. Tracheal synovial sarcoma (SS) is even an extremely rare entity. Diagnosis of tracheal SS can be achieved with chromosomal translocation studies along with immunohistochemistry. Margin-free resection is the gold standard treatment.

**Case presentation:**

We report a case of tracheal SS, which presented with stridor with a history of chronic cough and was diagnosed with a battery of clinical investigations and managed successfully with tracheal resection surgery. In histology, it may mimic Ewing’s sarcoma. Immunohistochemically, SS stains positive for cytokeratin, epithelial membrane antigen, vimentin, and S100. Chromosomal translocation t(X;18) (p11;q11) is found in almost all SS. This genetic signature is the gold standard diagnostic modality for these tumors.

**Conclusion:**

Diagnosis of tracheal synovial sarcoma is challenging because of the rarity of the disease and common presenting symptoms to other tracheal pathology and is supplemented with chromosomal translocation study along with histopathology and immunohistochemistry. Tumor coring before definite surgical resection facilitates lung perfusion in obstructive tracheal pathology. A multidisciplinary team approach for diagnosis and management along with long-term follow-up are warranted.

## Background

Soft tissue sarcoma of the head and neck region consists of less than 10% of all soft tissue sarcoma [1]. The heterogeneity of histology is responsible for a wide variety of clinical presentations. The management protocol includes surgery/radiotherapy/chemotherapy/combination therapy. Tracheal tumors are an uncommon group of tumors, accounting for less than 1 % of all malignant tumors [2], of which a small percentage are mesenchymal in origin. Less than 3% of synovial sarcoma (SS) arises in the head and neck region [3]. Tracheal SS is even rarer. There is a scarcity of data in the literature. Hence, the management of the tracheal soft tissue sarcoma and it’s outcome are not assessed accurately. Here, we report a case of primary tracheal monophasic SS and the diagnostic dilemma associated with it.

## Case presentation

**Fig. 1:**
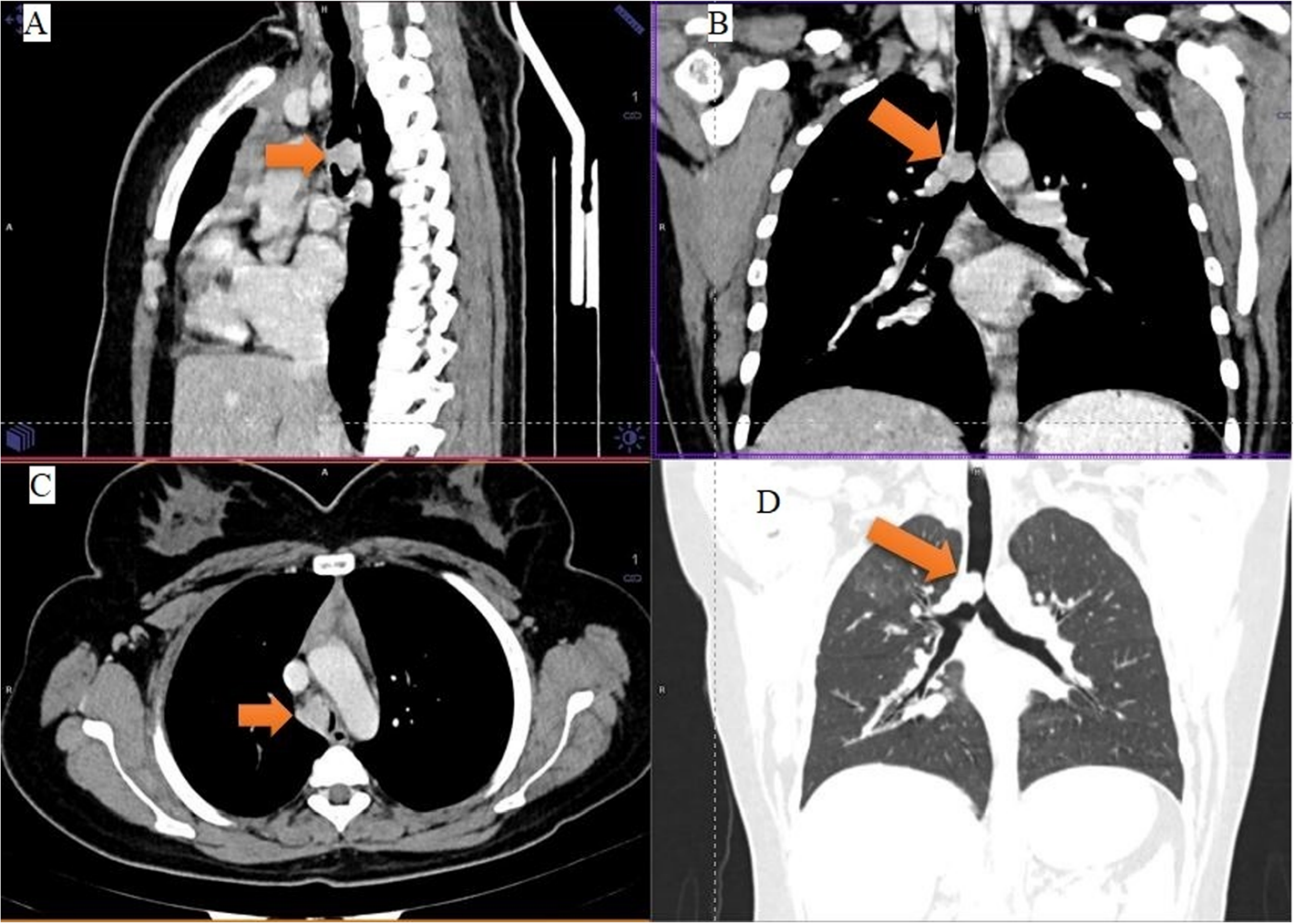
Sagittal (**a**), coronal (**b**), axial (**c**), and lung window (**d**) views of CT scans of thorax show a well-defined polypoidal soft tissue density lesion (solid yellow arrows) on the right posterolateral side of trachea at T4 vertebral level with gross luminal compromise, just proximal to carina with no extra luminal extension, laryngotracheal deviation, and lung collapse

A 26-year-old non-smoker female with no medical comorbidities and no relevant family history, who was having a cough for 3 months, presented to the emergency room with progressively increasing stridor. The patient was acyanotic and was having tachypnea with labored breathing. Chest X-ray was suggestive of right lung collapse. The computed tomography (CT) scan of neck and chest (done outside before presentation to our institute) demonstrated a 20 × 17 mm tracheal mass just proximal to the carina, on the right posterolateral side, occluding the tracheal lumen with no extraluminal extension. There was no regional lymphadenopathy or distant metastasis (Fig. 1a–d).

**Fig. 2:**
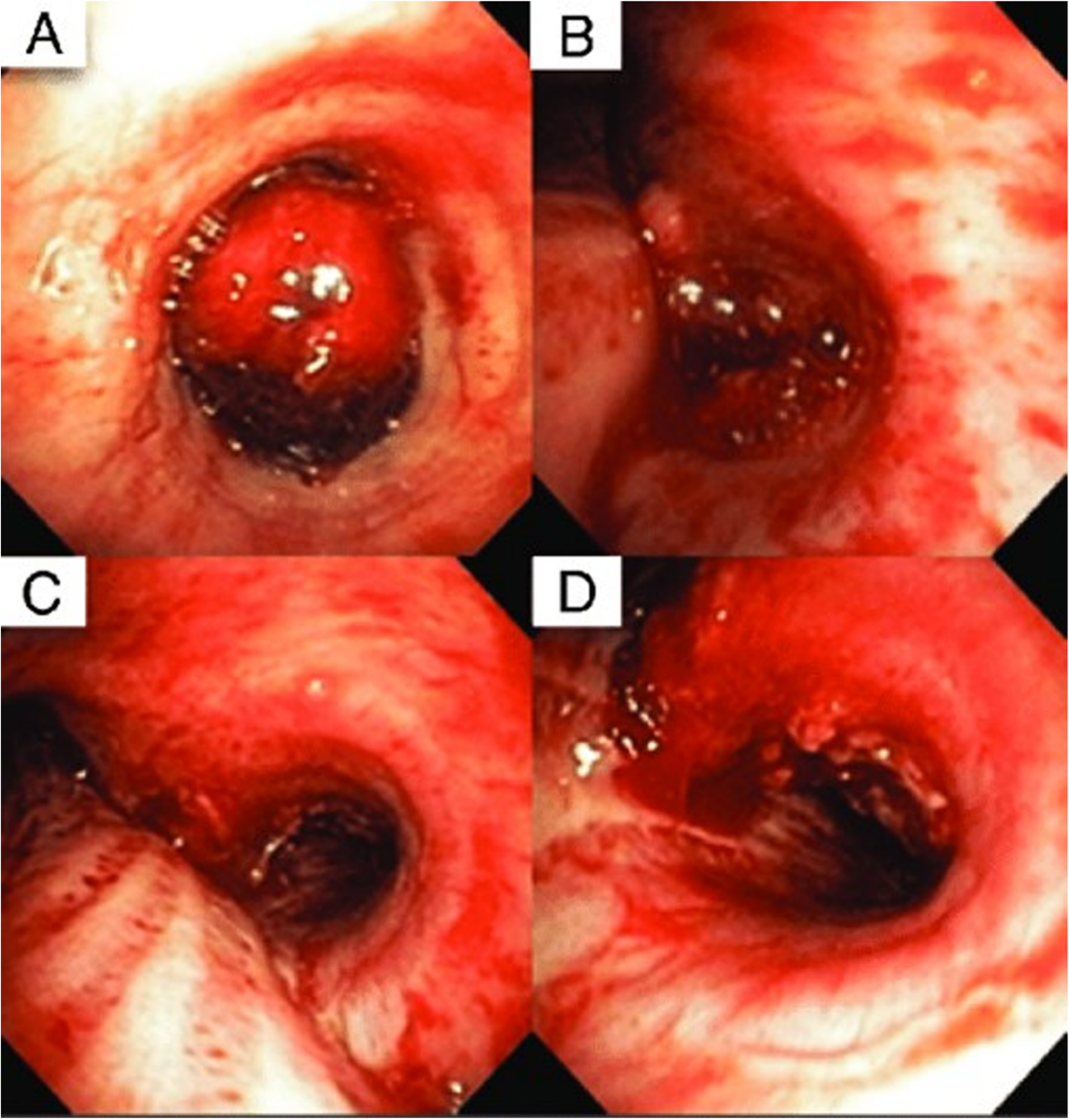
**a** Pre-debulking rigid bronchoscopy showing polypoidal obstructing growth, just proximal to the carina. **b**–**d** Post-debulking bronchoscopy showing residual tracheal tumor with normal distal tracheobronchial airways

Evaluation with fiber optic bronchoscopy (FOB) was done to assess the nature and extent of disease along with the feasibility of therapeutic debulking/coring. On bronchoscopy, there was an exophytic sessile obstructing ulcero-proliferative growth of size approximately 2 × 2 cm just proximal to the carina and extending for 2.5 cm above it. Tumor coring was facilitated with a rigid bronchoscope under sedation (Fig. 2a–d). Histopathology of the debulking specimen was suggestive of neuroendocrine carcinoma, being focally immunopositive for pancytokeratin and synaptophysin, while immune-negative for chromogranin and TTF-1. Ki-67 labeling index was 20–30% in the highest proliferation area.

After symptomatic improvement with debulking, the patient was planned for definitive surgery two weeks later. Vitals parameters were in the normal range. Her general and systemic examination findings were essentially normal, except for respiratory system evaluation; there were decreased breath sounds on the right side with focal crepitations. Hematological work-up was normal. Post-tumor debulking/coring, Pulmonary function test (PFT) demonstrated forced expiratory volume in 1 s (FEV1) and forced vital capacity (FVC) 2.07 L (75% of predicted) and 3.28 L (94% of predicted) respectively.

The surgery was planned through the right postero-lateral muscle-sparing thoracotomy approach along the fourth intercostal space using isolated left lung ventilation on total intravenous anesthesia. Intraoperatively, there was an ulcerative tumor of size 1.5 × 1.0 × 1.0 cm in the right posterolateral wall of the trachea, starting just proximal to the carina and extending 2 cm above it. There was no paratracheal extension. The patient underwent tracheal resection with subcarinal and paratracheal lymphadenectomy with tracheoplasty after inferior pulmonary ligament release and hilar mobilization. The trachea was adequately mobilized on anterior and posterior aspects to facilitate tension-free tracheoplasty and the tracheal defect of ~ 3 × 2 cm was closed in transverse fashion using 4–0 interrupted absorbable monofilament sutures. Primary tracheoplasty was augmented with the pericardial fat pad. After the procedure, check FOB was done to ensure airtight closure. The patient was discharged on the fifth day of surgery after uneventful recovery. There were no early or late surgical morbidities.

**Fig. 3:**
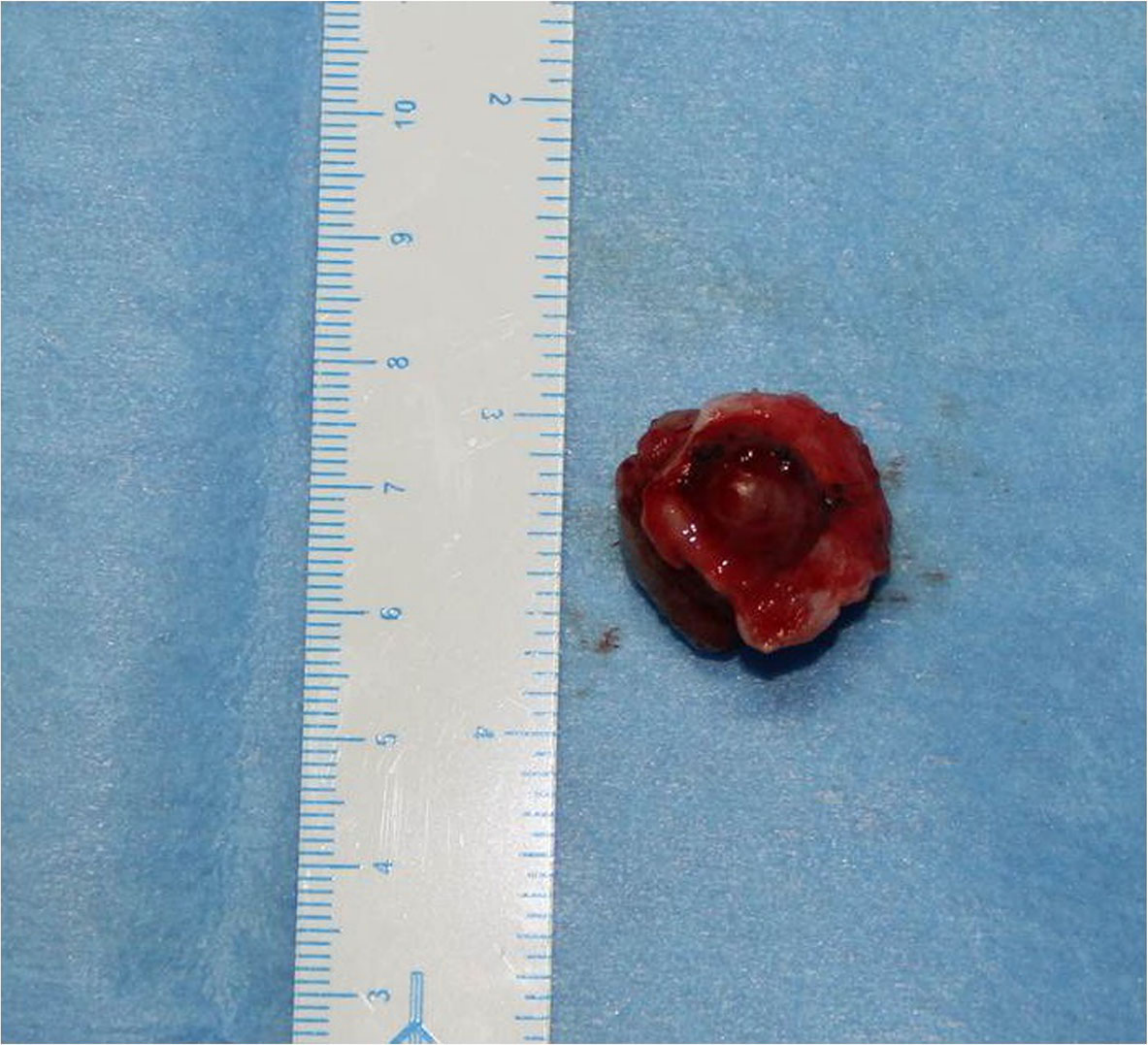
Gross specimen of tracheal tumor with tracheal cartilage

**Fig. 4:**
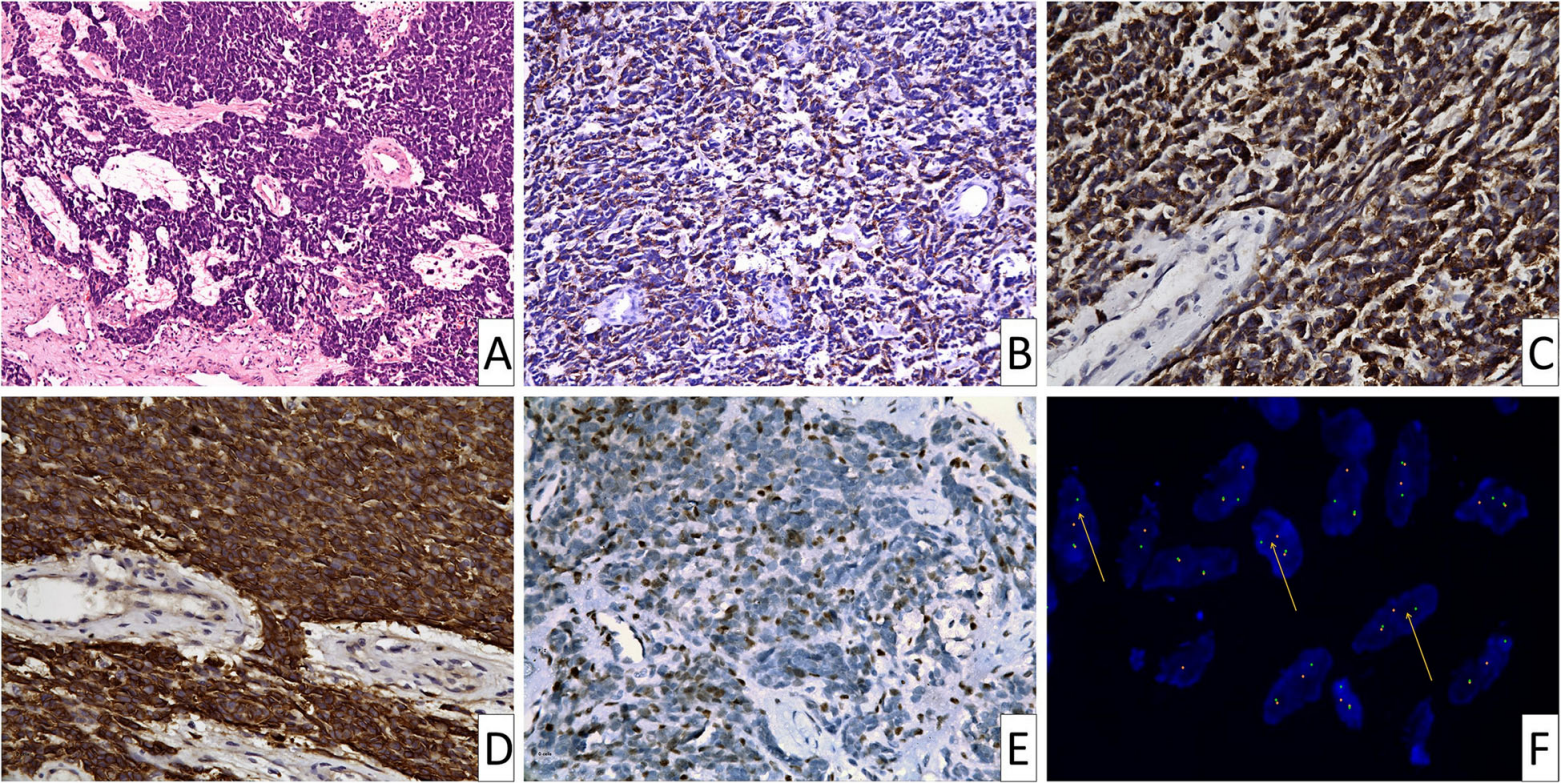
Microscopic pictures from tracheal mass shows a tumor composed of monomorphic small round cells arranged in sheets (**a**; H&E, 100×), tumor shows immunopositivity for epithelial membrane antigen (EMA) (**b**; 100×), bcl2 (**c**; 200×), MIC2 (**d**; 200×), and TLE1 (**e**; 100×). Interphase fluorescent in-situ hybridization for translocation (X;18) (SYT Vysis break-apart probe) shows a separation of the 5′ and 3′ SYT signals in many of the tumor cell nuclei (arrows)

Histopathological specimen comprised grossly of a fragment of the cartilage of size 1.5 × 1.0 cm with attached tumor tissue 1.5 × 1.0 cm (Fig. 3). The cut surface of the tumor was yellowish-white. Sections from the tumor revealed a small round cell tumor arranged in sheets. All resected margins (anterior, inferior, subcarinal, and right lower paratracheal) and dissected 3 lymph nodes adjacent to the main specimen were free of tumor. Immunohistochemically, tumor cells were immune-positive for EMA, BCL2, MIC-2, and TLE1, while immune-negative for TTF-1, chromogranin, desmin, and myogenin. MIB-1 index was around 50–60%. Overall morphological and immune-histochemical features were favoring synovial sarcoma with a differential diagnosis of Ewing’s sarcoma (ES). Fluorescence in situ hybridization (FISH) analysis for SS demonstrated dual-color break-apart rearrangement probe (Vysis) which indicates t(X;18)(p11.2;q11.2), reciprocal translocation between SS18 gene on chromosome 18 (18q11.2), and SSX gene on chromosome X (Xp11.2) causes the presence of SS18-SSX fusion gene (Fig. 4). FISH analysis for ES did not show dual-color break-apart rearrangement probe (Vysis) which indicates the absence of t(11;22)(q24;q12), translocation between EWSR1 gene on chromosome 22 (22q12), and FLI-1 gene on chromosome 11 (11q24) causes the EWSR1/FLI-1 fusion gene. Based on these histological, immunohistochemical, and molecular genetic analyses, a final diagnosis of synovial sarcoma of the trachea was considered.

**Fig. 5:**
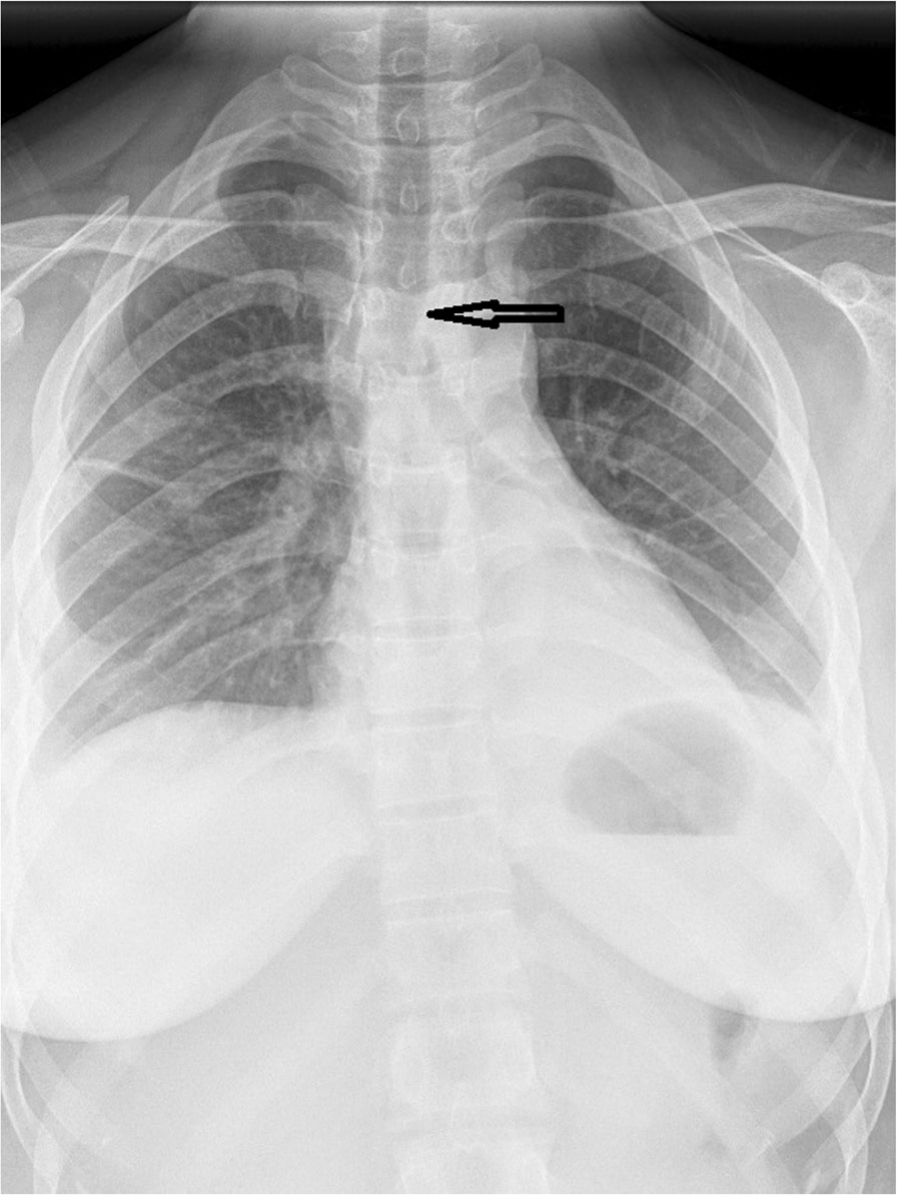
Chest X-ray showing normal post-operative change in trachea (black arrow) with no lung metastasis

Because of small-sized primary tracheal synovial sarcoma with negative margins dissection, no adjuvant treatments were advised in a multidisciplinary tumor board meeting. The patient was kept on follow up with clinical examinations and serial chest X-ray and FOB. One year after surgery, there was locoregional control and had no distant metastases in chest X-ray imaging (Fig. 5).

## Discussion

Primary tracheal SS is extremely rare. A couple of case reports of primary tracheal SS are reported in the English literature [4, 5]. Corrales et al. did a surgical resection of primary cervical synovial sarcoma and primary anastomosis assisted by laryngeal-releasing maneuvers, without any complication. The author confirmed the diagnosis by demonstrating the SYT-SSX gene rearrangement and ascertained that surgery was the first-line treatment for such malignancy. Sykes et al. did en bloc tumor excision with trachea through a low collar incision by the neck approach and primary end-to-end tracheal anastomosis was performed. The patient received a total dose of 62 Gy external beam irradiation in 22 fractions of 180 cGy and boost therapy in 12 fractions of 180 cGy. No adjuvant chemotherapy was administered to this patient. The author concluded that surgery was the optimal treatment modality. The treatment outcome was good in the above-mentioned literature with no evidence of locoregional disease recurrence or distant metastasis. Likewise, our patient is also disease-free until the last follow-up date. These tumors grow intraluminally and are diagnosed only after a significant portion of the lumen is compromised.

Synovial sarcoma should be excluded from Ewing’s sarcoma. SS is a clinically distinct entity. It is reclassified into three sub-types: monophasic, biphasic, and poorly differentiated variants [6]. Poorly differentiated sub-type has aggressive clinical behavior. MIC2 gene expression is usually found in ES [7]. Chromosomal translocations t(11;22) (q24;ql2) and t(21;22) (q22;ql2) are considered to be specific for PNET/ES, while t(X;18) (p11;q11) translocation is specific for SS [7]. Hence, immunohistochemical and genotypic analyses are considered to be effective diagnostic tools.

There is no specific staging system for tracheal sarcoma, because of a small number of cases with mixed histology and insufficient information about survival. So, there are no clear guidelines for diagnosis and management. Optimal treatment is en bloc surgical resection with negative margins. Debulking before the definite surgical resection is required to improve respiratory distress. It is an effective and safe procedure for airway obstruction due to tracheobronchial tumors [8]. The histology of debulking specimen in the present case renders the diagnosis of neuroendocrine carcinoma (NEC). The most common neuroendocrine carcinoma is carcinoid tumor and it has low malignant potential and has a comparatively good prognosis. Microscopically, these tumors have large polyhedral cells with hyperchromatic nuclei

Often anaplastic cells, areas of necrosis and high mitotic figures are also seen. Tumor cells are usually immunopositive for chromogranin, synaptophysin, pancytokeratin, CEA, calcitonin, and p53, while immunonegative for TTF-1 [9].

Nevertheless, the surgical management of lower tracheal tumor is always a challenge given difficult access, being surrounded by vital organs. In general, adjuvant chemoradiotherapy is advocated for high-grade sarcoma. The prognosis of head and neck SS depends on tumor grade, extent, and size. But because of the rarity of tumor, there are no definite protocols for adjuvant treatment and follow-up. Histopathological diagnosis is a challenge to differentiate between synovial sarcoma and Ewing’s sarcoma, which may mimic each other. The value of FISH translocation studies to make a specific diagnosis cannot be overemphasized. In literature, local recurrence and distant metastasis in head and neck sarcomas are reported as late as 62 months after surgery, so long-term follow-up with serial imaging and FOB is indicated in such rare aggressive tumor [10]. Six monthly follow-up was advised for the first five years after surgery and thereafter annually.

## Conclusion

Tracheal SS needs stringent clinical diagnostic evaluation for attaining a definite diagnosis. The clinical symptoms may be misleading. Histology, immunohistochemistry, FISH analysis for chromosomal translocation studies are vital for accurate diagnosis of this rare clinical entity. A multidisciplinary approach for diagnosis and management along with long-term follow-up is always desirable. Targeting the characteristic chromosomal translocation t(X:18) helps in making a specific diagnosis and further management of this tumor.

## Data Availability

All data generated or analyzed during this study had already been included in this manuscript. Patient’s case file was retrieved from the medical record section of the institution. The clinical data had been collected from the prospectively maintained computerized database and the case file. The follow-up status was updated from the above-mentioned manner.

## Abbreviations

CT: Computed tomography
ES: Ewing’s sarcoma
FEV1: Forced expiratory volume in 1 s
FISH: Fluorescence in situ hybridization
FOB: Fiber optic bronchoscopy
FVC: Forced vital capacity
PFT: Pulmonary function test
SS: Synovial sarcoma
